# The expanding role of hormones in cancer

**DOI:** 10.1530/EO-21-0001

**Published:** 2021-02-04

**Authors:** Edward P Gelmann

**Affiliations:** 1 UArizona Cancer Center, University of Arizona, Tucson, Arizona, USA

In launching a new journal that is endorsed by as austere a group as the Society for Endocrinology, one is tempted at first to cite the opening words of Dickens’ *Tale of Two Cities*. We live in an age of instantaneous communication and immediate dissemination of information without assurances or safeguards for the veracity of what we learn. This is also a time of technical capabilities, which exceed our capacity to use them fully, in order to analyze biological phenomena and probe the mechanisms of disease. There are many more questions and problems that can be answered in a timely manner, had we only the resources to do so. Nevertheless, discovery is proceeding at an expanding pace, and the need for rapid publication and wide dissemination after thorough and responsible review has never been greater. An open access, online journal serves both readers and investigators in this age of preprint servers, Twitter announcements, email, and blogs.

Cancers of endocrine and endocrine-responsive organs represent a large and growing health burden in the world. Certainly, the most prevalent are breast and prostate cancers, both more common in economically advanced countries. As economic advancement benefits more and more of the world’s population, both these diseases are sure to increase in incidence over the coming decades. Changes in diet, improvements in childhood and adolescent health, decreased incidence of infectious disease, control of HIV, and decreased cardiovascular mortality will all contribute to increased incidence of both breast and prostate cancers in many countries. Both prevention and treatment of these two cancers have benefitted from the development of new drugs and predictive markers during the last two decades. Moreover, since, to date, novel immunotherapies have found limited applicability to both breast and prostate cancer, their unique dependence on hormone-regulated pathways will likely be the focus of further diagnostic, preventive, and therapeutic approaches for some time to come.

Interactions of hormones with their receptors are also frequent agonists in the carcinogenic process. Our understanding of the mechanisms of hormone-mediated molecular carcinogenesis is still far from complete. For example, the mechanism by which *BRCA2* heterozygosity favors the development of breast, prostate, and ovarian cancers is far from well understood. The role of pharmacologic hormones in cancer causation and antihormones in cancer prevention both beg better understanding for further improvement in clinical application and drug design. Hormonal carcinogenesis may, additionally, possibly be caused by hormonal mimics from nonphysiologic sources. The literature is replete with reports of both inorganic and organic molecular contaminants that can act as receptor antagonists. This area of the investigation remains ripe for further investigation and mechanistic resolution. Additionally, diet itself, so often impugned in carcinogenesis, but so evasive regarding experimentation, will come into greater focus. It is hard to ignore diet and obesity, since the latter is one of the undeniable causes of endometrial cancer.

A wealth of future findings lies dormant in the public domain, where a high tide of gene sequencing and expression data lie awaiting insight and analysis. It is not hard to predict that genetic aberrations and polymorphisms will be found that influence both the causes and treatment of hormone-responsive cancers. Commercial entities have developed empirical panels of gene expression that serve as prognostic and predictive markers without an obvious link to disease mechanisms. Perhaps in the future, prognostic and predictive tools will be found directly relating to mechanisms related to the regulation of hormonal signaling. Certainly, endocrine cancers result from well-known hereditary mutations. Much more remains to be clarified about the selectivity of those genetic lesions and how they may best be exploited for prevention and treatment.

All hormones affect gene action that is influenced by chromatin modification, gene splicing, and regulatory RNAs. The complexity of gene expression and its pathogenic aberrations will long be the subject of investigation for a better understanding of the behavior and treatment of neoplasia. Drugs that affect chromatin modification are in use for the treatment of hematologic malignancies. But their limited use in solid tumors may only imply we have not found the right targets and the right agents for clinical application to solid tumors. We also hope to see a better understanding of the role of regulatory RNA in disease behavior and perhaps the identification of candidate RNA species that have clinical utility.

Cell surface receptors are unique markers for some malignant cells. Major therapeutic advances have also come from the targeting of hormone receptors and other cell surface molecules in order to deliver cytotoxic agents to the cells expressing those proteins. The chemistry that allowed the binding of toxic radioisotopes to antibodies or synthetic ligands has now resulted in clinical reagents that impact survival in neuroendocrine cancers, for example. It is likely that many more targets that can be exploited for therapeutic delivery will be described in the coming years.

*Endocrine Oncology* seeks to play an important role in the landscape of scientific ideas, discoveries, and advances for the spectrum of endocrine malignancies. We are committed to scientific rigor, rapid turn-around, relevance, and the advancement of knowledge. We look forward to working with the scientific community.

